# Case Report: Vincristine-induced acute pancreatitis in pediatric Wilms tumor: first reported case challenging previous risk classifications and proposing vigilant monitoring protocols

**DOI:** 10.3389/fped.2025.1636941

**Published:** 2026-01-12

**Authors:** Ting Liu, Zhongqiang Cao, Fangyuan Lai, Huanli Xu, Zebin Chen, Xiuli Yuan, Xiaoya Liu

**Affiliations:** 1 Department of Pharmacy, Shenzhen Children’s Hospital, Shenzhen, China; 2 Department of Hematology and Oncology, Shenzhen Children's Hospital, Shenzhen, China

**Keywords:** adverse drug reaction, drug-induced pancreatitis, pancreatitis, pediatric, wilms tumor

## Abstract

Vincristine, a cornerstone vinca alkaloid in pediatric oncology, has historically been regarded as a low-risk agent for drug-induced acute pancreatitis (DIAP). We report the first documented pediatric case of vincristine monotherapy-associated acute pancreatitis, challenging existing toxicity paradigms. A 3.25-year-old boy with stage IV Wilms tumor developed acute-onset fever and localized periumbilical pain 48 h after vincristine infusion (0.9 mg), administered within standard dosing. Laboratory testing confirmed marked pancreatic hyperenzymemia (serum α-amylase 2,022 U/L, 12.5 × ULN; lipase 568 U/L, 7.1 × ULN), and contrast-enhanced ultrasonography revealed pancreatic edema. A Naranjo score of 7 indicated *probable* causality, further supported by complete symptom resolution after substitution with vindesine and no recurrence during six months of follow-up. Genetic testing showed no CEP72 (rs924607) susceptibility variant, suggesting a hypersensitivity rather than pharmacogenomic mechanism. This case highlights the need to re-evaluate vincristine's DIAP risk profile in children and supports implementing routine post-infusion monitoring of pancreatic enzymes in high-risk patients. Vindesine may serve as a safe and effective alternative agent. These findings underscore the importance of updating pediatric chemotherapy safety guidelines for vinca alkaloid-containing regimens.

## Introduction

Vincristine is a widely used vinca alkaloid that acts by disrupting microtubule assembly, thereby inhibiting cell division and tumor growth. Although generally well tolerated, its known toxicities include peripheral neuropathy, myelosuppression, and gastrointestinal disturbances (1). Acute pancreatitis (AP) is an inflammatory condition characterized by pancreatic autodigestion and carries significant morbidity and mortality (2). While multiple chemotherapeutic agents, such as pegaspargase, cytarabine, and mercaptopurine, have been implicated in AP (3–5), vincristine-induced AP is exceedingly rare and has not been previously reported in children.

Given the widespread use of vincristine in pediatric oncology, reporting suspected cases of vincristine-associated AP is essential for improving risk recognition, diagnostic accuracy, and patient safety. This report presents the first pediatric case of AP associated with vincristine monotherapy and discusses its potential mechanisms, diagnostic considerations, and clinical implications.

## Case presentation

A 3.25-year-old boy presented with a left abdominal mass without abdominal pain, fever, vomiting, or bleeding. Imaging and biopsy confirmed left-sided Wilms tumor with pulmonary metastases. Baseline abdominal Doppler ultrasonography showed normal liver, gallbladder, bile ducts, pancreas, and vasculature.

Preoperative chemotherapy was initiated according to the SIOP–RTSG 2016 protocol (6), consisting of vincristine (1.5 mg/m^2^ on Day 1 of Weeks 1–6), doxorubicin (50 mg/m^2^ on Day 1 of Weeks 1 and 5), and dactinomycin (45 µg/kg on Day 1 of Weeks 1, 3, and 5). The patient tolerated the first week well. Although the vincristine was scheduled for day 8, the child returned one day later than planned due to long-distance travel and therefore received 0.9 mg vincristine intravenously on day 9. He was discharged afterward but returned two days later with fever (39.2℃) and persistent periumbilical abdominal pain without vomiting.

**Figure 1:**
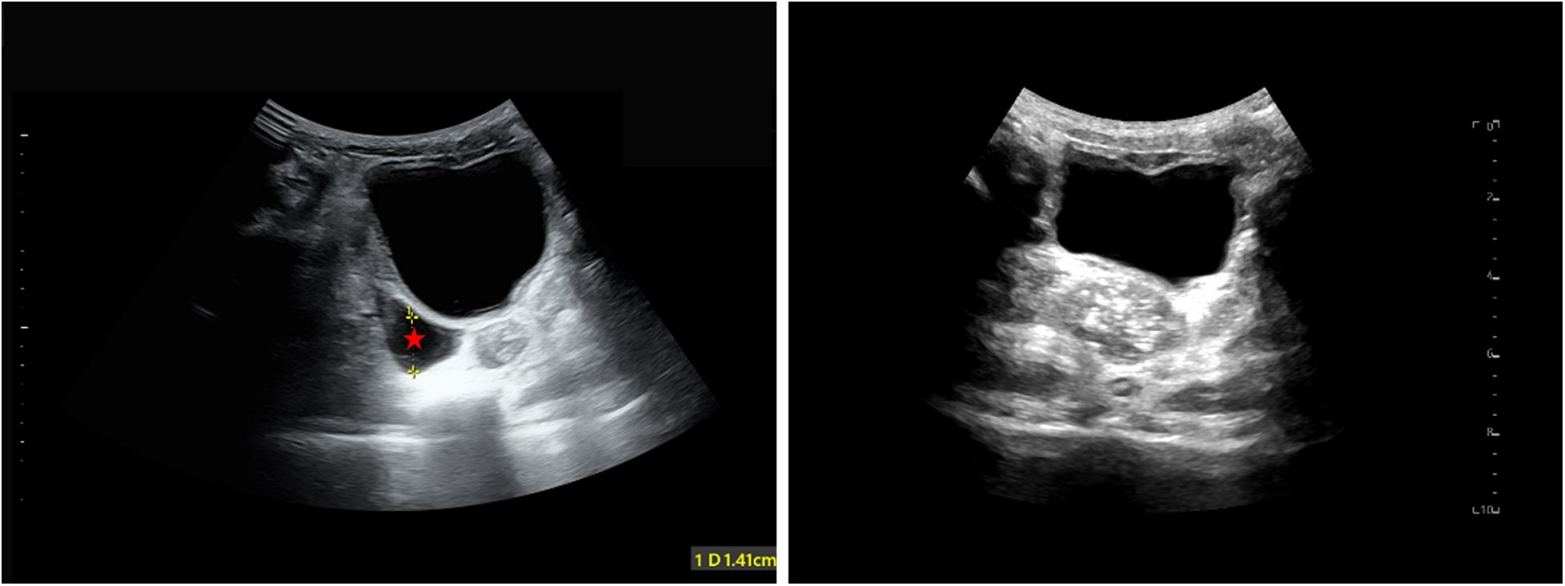
Doppler ultrasonography revealed a small amount of fluid in the lower abdomen with a maximum depth of 1.4 cm (left, red star), which was no longer present after treatment (right).

Laboratory tests revealed marked elevation in pancreatic enzymes: serum amylase rising to 2,022 U/L (normal: 25–101 U/L) and urinary amylase to 2,356 U/L (normal: 0–1,200 U/L). Abdominal ultrasonography showed a small amount of peritoneal fluid (Figure 1) without abnormalities in the liver, gallbladder, spleen, or pancreas. No significant changes in bowel habits were noted during the chemotherapy period.

Typhlitis was considered unlikely because the absolute neutrophil count remained above 2.0 × 10^9^/L and there were no features of neutropenic enterocolitis. Infection was ruled out based on normal white blood cell, procalcitonin levels and negative testing for respiratory viruses, despite the presence of fever (7). Based on clinical findings and Doppler ultrasonography, a diagnosis of acute pancreatitis (AP) was established. Although AP has been reported in adults receiving multidrug chemotherapy, vincristine is usually considered the least likely causative agent. In this case, a Naranjo score of 7 supported probable vincristine-induced AP (Table 1).

**Table 1:** Naranjo adverse drug reaction probability scale for vincristine-induced acute pancreatitis (14).

Question	Yes	No	Do not know	Score
1. Are there previous conclusion reports on this reaction?	+1	0	0	+1
2. Did the adverse event appear after the suspected drug was administered?	+2	−1	0	+2
3. Did the adverse reaction improve when the drug was discontinued or a specific antagonist was administered?	+1	0	0	+1
4. Did the adverse reaction reappear when the drug was readministered?	+2	−1	0	0
5. Are there alternative causes that could on their own have caused the reaction?	−1	+2	0	+2
6. Did the reaction reappear when a placebo was given?	−1	+1	0	0
7. Was the drug detected in blood or other fluids in concentrations known to be toxic?	+1	0	0	0
8. Was the reaction more severe when the dose was increased or less severe when the dose was decreased?	+1	0	0	0
9. Did the patient have a similar reaction to the same or similar drugs in any previous exposure?	+1	0	0	0
10. Was the adverse event confirmed by any objective evidence?	+1	0	0	+1

The total score ≥9 indicates that the correlation between the drug and adverse reactions is positive; The total score of 5∼8 indicates the correlation between the drug and adverse reaction is probably related; The total score of 1∼4 indicates the correlation between the drug and adverse reaction is possibly related; The total score≤0 indicates the correlation between the drug and adverse reaction is suspicious.

**Figure 2:**
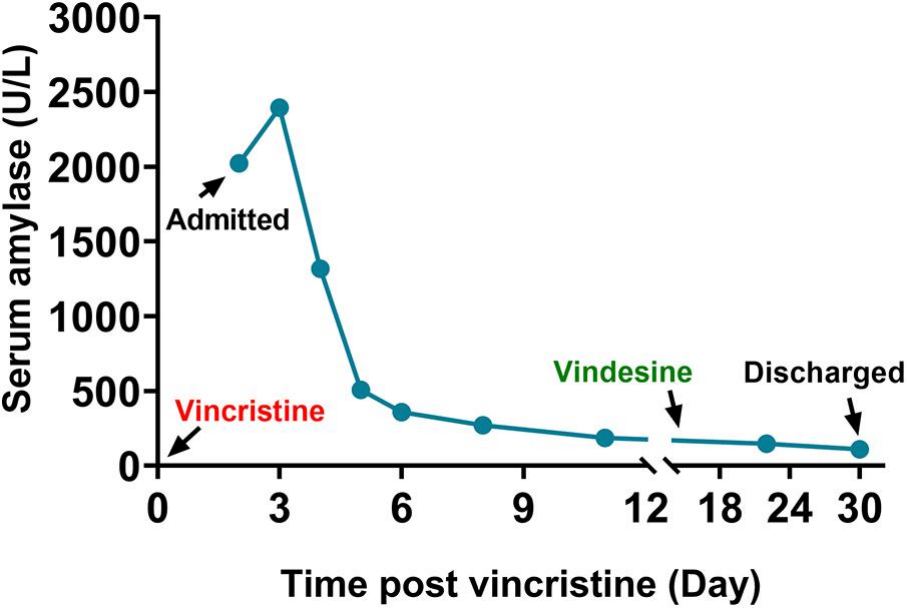
Relationship between changes in amylase levels and the timing of vincristine administration during hospitalization. Arrows indicate the timing of subsequent chemotherapy drug administration.

Supportive management was initiated, including fasting, intravenous nutrition (∼40 kcal/kg/day, total volume 720 mL), octreotide, ulinastatin, and omeprazole to suppress pancreatic stimulation. By Day 13, the child's fever had resolved, abdominal pain had subsided, and amylase levels began to decline. By Day 15, abdominal pain had completely resolved and serum amylase had decreased to 269 U/L (Figure 2). A liquid diet was reintroduced. Octreotide and parenteral nutrition were maintained until sustained enzyme decline and symptom resolution, after which enteral feeding resumed. Two follow-up ultrasounds showed no pancreatic abnormalities. CEP72 genotyping revealed no pathogenic variant.

Given the suspected association with vincristine, the chemotherapy regimen was modified by substituting vindesine. Over two subsequent triple-drug cycles and additional vindesine monotherapy, no recurrence of AP occurred.

## Discussion

Vincristine is a key chemotherapeutic agent for pediatric malignancies such as leukemia, lymphoma, and certain solid tumors (8). However, vincristine carries notable toxicity risks, largely attributable to its effects on nervous tissue and rapidly dividing cells. Neurotoxicity is the most prominent adverse reaction, typically manifesting as peripheral neuropathy characterized by numbness, tingling, muscle weakness, or motor dysfunction. Additional toxicities include mild myelosuppression, increased susceptibility to infections, and gastrointestinal disturbances (1, 9).

AP is a rare but serious adverse effect of vincristine, with only a few cases documented in adults. In two large reviews, vincristine and vinblastine were classified among the chemotherapeutic agents with the lowest risk of inducing AP (10, 11). Socinski et al. (12) reported two patients with germ cell tumors who developed AP while receiving cisplatin, bleomycin, and vinblastine. Another case described a 64-year-old woman who developed AP during treatment with a multidrug regimen containing vincristine, doxorubicin, and dexamethasone, in which vincristine was considered the least likely contributor (13). The scarcity of clinical reports may lead to underestimation of vincristine-induced AP, particularly in children, where abdominal pain is a frequent symptom during chemotherapy and may obscure early recognition of pancreatitis.

In this case, vincristine received a Naranjo score of 7, indicating probable causality (Table 1) (14). First, abdominal pain developed two days after vincristine administration, demonstrating a clear temporal association. Second, alternative etiologies, such as alcohol use, gallstones, hypercalcemia, hyperlipidemia, and hereditary factors, were excluded through physical examination, biochemical testing, and imaging studies. Third, both abdominal pain and pancreatic enzyme elevations resolved during the subsequent treatment cycle once vincristine was discontinued. Taken together, these findings support vincristine as the most likely causative agent of AP in this patient. To the best of our knowledge, this represents the first reported case of vincristine-induced AP in a child.

Drug-induced AP (DIAP) is the second most common etiology of AP in children after biliary causes (15). Evidence is limited, and mechanisms remain incompletely understood. Proposed pathways include immune-mediated reactions, idiosyncratic responses, direct toxicity, accumulation of toxic metabolites, sphincter of Oddi spasm, and genetic susceptibility (7, 16, 17).

The time from drug initiation to the onset of pancreatitis varies according to the underlying mechanism. Immune-mediated adverse drug reactions can induce pancreatitis within weeks of starting therapy, whereas pancreatitis resulting from toxic metabolites typically manifests after several months of use. Hypersensitivity-related AP generally develops within hours to days after drug administration (18). In our patient, hypersensitivity was considered the most likely mechanism. First, the patient experienced abdominal pain just two days after receiving vincristine. Second, the administered dose of vincristine was 0.9 mg, consistent with the recommended dosage, thereby excluding overdose as a contributing factor. This supports a hypersensitivity mediated mechanism, which is typically not dose-dependent.

Direct cytotoxicity was also considered a potential mechanism. Animal studies have shown that vinca alkaloids can cause degeneration of pancreatic acinar cells (19). However, vincristine administration in dogs has not been associated with clinical pancreatitis (20). Genetic predisposition was further evaluated. Reduced expression of the CEP72 gene has been linked to increased vincristine sensitivity and heightened risk of toxicity (9). In this patient, genetic testing revealed a CEP72 genotype of CC (rs924607 C>T), representing a normal genotype. In conclusion, hypersensitivity appears to be the most plausible mechanism underlying vincristine-induced AP in this patient.

Rechallenge with vincristine was avoided for ethical reasons. Vincristine was successfully substituted with vindesine, with no recurrence of AP. Structural differences between the two agents may explain differential toxicity (21). Further research is needed to investigate pharmacokinetics, drug concentrations, and genetic factors that may influence pancreatic toxicity in children receiving vinca alkaloids.

In conclusion, this is the first case report of AP caused by vincristine in pediatric. This case describes a suspected instance of vincristine-induced AP in children with Wilms tumor, with the causality between vincristine and AP deemed probable. While AP caused by vincristine is a rare adverse effect, it can be life-threatening in some cases. Raising awareness of this potential risk is essential. Clinicians and pharmacists should remain vigilant for this adverse drug reaction, closely monitor clinical symptoms, and track key indicators such as amylase and lipase levels. Early detection and prompt intervention are crucial to ensuring the safety of chemotherapy in children.

## Patient perspective

The Shenzhen Children's Hospital's Medical Group reviewed and approved all studies involving human participants. The patient's legal guardian provided written informed consent for the publication of this case report.

## Data Availability

The original contributions presented in the study are included in the article/Supplementary Material, further inquiries can be directed to the corresponding authors.
